# Cryopreservation of mouse resources

**DOI:** 10.1186/s42826-020-00066-w

**Published:** 2020-09-17

**Authors:** Toru Takeo, Satohiro Nakao, Yoshiko Nakagawa, Jorge M. Sztein, Naomi Nakagata

**Affiliations:** 1 Division of Reproductive Engineering, Center for Animal Resources and Development, Institute of Resource Development and Analysis, Kumamoto University, 2-2-1 Honjo, Kumamoto, 860-0811 Japan; 2 Division of Reproductive Biotechnology and Innovation, Center for Animal Resources and Development, Institute of Resource Development and Analysis, Kumamoto University, Kumamoto, Japan

**Keywords:** Genetically engineered mice, Mouse bank, Reproductive technology, Sperm, Embryo, Cryopreservation, In vitro fertilization, Cold storage, Hands-on workshop

## Abstract

The cryopreservation of sperm and embryos is useful to efficiently archive valuable resources of genetically engineered mice. Till date, more than 60,000 strains of genetically engineered mice have been archived in mouse banks worldwide. Researchers can request for the archived mouse strains for their research projects. The research infrastructure of mouse banks improves the availability of mouse resources, the productivity of research projects, and the reproducibility of animal experiments. Our research team manages the mouse bank at the Center for Animal Resources and Development in Kumamoto University and continuously develops new techniques in mouse reproductive technology to efficiently improve the system of mouse banking. In this review, we introduce the activities of mouse banks and the latest techniques used in mouse reproductive technology.

## Introduction

A genetically engineered mouse is a powerful tool to elucidate the complex communications between genes or organs in health and diseases [1]. Moreover, humanized mouse models derived from immunosuppressed mice are helpful to bridge the gap of discovery and the development of new medicines between human and animal experiments [2]. Therefore, it is important to enhance the availability and accessibility of mouse resources to conduct research projects efficiently using valuable mouse models.

A mouse bank plays a vital role in archiving and supplying mouse resources [3]. In Kumamoto University, the Center for Animal Resources and Development (CARD) was established as a research center for genetics and biomedical science using genetically engineered mice and as a quality center of mouse resources as a mouse bank in 1998 [4, 5]. The CARD provides services of production, cryopreservation, and supply of genetically engineered mice and established a searchable database of the archived mouse strains known as the CARD Resource Database (CARD R-BASE, http://cardb.cc.kumamoto-u.ac.jp/transgenic/). Till date, the international collaboration of mouse banks (International Mouse Strain Resource: IMSR) has successfully collected more than 60,000 strains of genetically engineered mice (Table 1). The archived mouse strains can be browsed through the IMSR website (http://www.findmice.org/) [6]. Researchers can obtain live mice, cryopreserved embryos, or the sperm of choice from those mouse banks.

**Table 1:** Mouse Banks (date as of August 4, 2020)

Repository	Region	Mouse strain
Australian Phenome Bank (APB)	Australia	1619
Animal Resources Centre (ARC)	Australia	16
Center for Animal Resources and Development (CARD)	Japan	1757
Cornell Heart Lung Blood Resource for Optogenetic Mouse Signaling (CHROMus)	USA	8
Canadian Mouse Mutant Repository (CMMR)	Canada	845
Charles River Laboratories (CRL)	USA	56
Cystic Fibrosis Mouse Model Core (CWR)	USA	10
Dr. Elizabeth M. Simpson, Ph.D. (EMS)	Canada	4
European Mouse Mutant Archive (EMMA)	Germany	7062
genOway (GENO)	France	9
GemPharmatech (GPT)	China	9889
MRC Harwell (HAR)	UK	1491
JAX Mice and Services (JAX)	USA	11,161
Korea Mouse Phenotyping Center (KMPC)	Korea	157
Mutant Mouse Regional Resource Centers (MMRRC)	USA	17,223
MUGEN Mouse Database (MUGEN)	Greece	75
National Cancer Institute at Frederick (NCIMR)	USA	139
National Institute of Genetics (NIG)	Japan	142
Oriental BioService, Inc. (OBS)	Japan	29
Oak Ridge Collection at JAX (ORNL)	USA	908
RIKEN BioResource Research Center (RBRC)	Japan	5357
National Applied Research Laboratories (RMRC-NLAC)	Taiwan	351
Shanghai Model Organisms Center, Inc. (SMOC)	China	2954
Taconic Biosciences (TAC)	USA	2725
Texas A&M Institute for Genomic Medicine (TIGM)	USA	195
University of North Carolina, Chapel Hill - Systems Genetics Core (UNC)	USA	75
	Total	64,257

In Asia, an international association of mouse research centers and mouse banks known as the Asian Mouse Mutagenesis and Resource Association (AMMRA, http://ammra.info/) was organized and has been functioning since 2006 [7]. The AMMRA aims at producing original mouse resources and promoting international collaboration in Asia. At the AMMRA conference, strategies are discussed to improve science using our resources, technology, and network, and workshops are held to educate students, technicians, and young researchers. Furthermore, the AMMRA participates in the Global Mouse Models for COVID-19 Consortium to support research fighting the coronavirus pandemic.

In a mouse bank, reproductive technology plays key roles in the efficient production, preservation, and transport of genetically engineered mice. Our center continuously refines the mouse reproductive technology to enhance the function of the mouse bank system. Till date, we have overcome several problems in mouse reproductive technology and have efficiently archived mouse resources by sperm and embryo cryopreservation to produce eggs and embryos using the techniques of ultrasuperovulation and in vitro fertilization and to establish the worldwide shipment of cryopreserved or cold-stored embryos and sperm [5]. Our techniques are used widely in mouse repositories and transgenic facilities [8–11]. In this review, we introduce the latest techniques used in the CARD mouse bank.

## Mouse reproductive technology

### Sperm cryopreservation

Sperm cryopreservation is the most cost-effective method to preserve mouse strains [12, 13]. Cryopreserved sperm can be preserved permanently in a liquid nitrogen tank and animals can be reproduced using in vitro fertilization and embryo transfer techniques. Potentially, more than 2000 pups can be produced from the cryopreserved sperm collected from a male mouse. Cryopreserved sperm can be transported in a dry shipper at − 196 °C or a shipment box containing dry ice at − 79 °C [14]. Prof. Nakagata developed the fundamental system of mouse sperm cryopreservation using a cryoprotectant composed of 18% raffinose pentahydrate and 3% skim milk (Nakagata method) [15].

However, there was a critical problem concerning the low fertility (0–20%) of cryopreserved sperm in C57BL/6 mice [16, 17]. To overcome this problem, we improved the raffinose- and skim-milk-based cryoprotectant by adding 100 mM l-glutamine (modified R18S3) [18]. We also developed a system of in vitro fertilization using frozen–thawed sperm to enhance the fertilization rate by treating with methyl-β-cyclodextrin (MBCD) and reduced glutathione (GSH). During sperm preincubation, MBCD (0.75 mM) increased the fertilization rate of frozen–thawed mouse sperm by stimulating cholesterol efflux from the sperm membrane [19]. In the fertilization medium, 1.0 mM of GSH or cysteine analogs supported sperm penetration through the zona pellucida and increased the fertilization rate by dissecting the disulfide bonds of the zona pellucida [20, 21]. Combining these techniques, we developed an optimized protocol for the cryopreservation of mouse sperm and in vitro fertilization using the frozen–thawed sperm [22]. A review describing the history of technology development in mouse sperm cryopreservation was written by Prof. Sztein [23].

### Embryo and oocyte vitrification

The vitrification of mouse embryos is useful to preserve mouse resources and readily reanimate homozygote mutant mice [24, 25]. Vitrified embryos can be preserved permanently in a liquid nitrogen tank at − 196 °C [26]. A standardized protocol in mice consists of a simple vitrification method using 1 M dimethyl sulfoxide (DMSO) and a mixture of 2 M DMSO, 1 M acetoamide, and 3 M propanediol (DAP213) used as the vitrification solution [27]. More than 90% of vitrified–warmed embryos can survive and 30–50% of the survived embryos can develop into pups via embryo transfer.

The vitrification of mouse oocytes is helpful for the emergent use of in vitro fertilization when there is a shortage of oocytes owing to superovulation failure or the delayed transport of cold-stored sperm. The simple vitrification method is also applicable to the cryopreservation of mouse oocytes [28, 29]. However, it has been observed that the prolonged exposure of hyaluronidase to remove cumulus cells from oocytes decreased the fertilization rate of cryopreserved mouse oocytes [30]. Treatment with N-acetyl cysteine (NAC) was found to recover the fertilizing ability of vitrified–warmed mouse oocytes by alleviating zona hardening [31].

The vitrification of mouse oocytes in the pronuclear stage was found to be useful for the production of genetically modified mice by genome editing techniques. Fertilized oocytes were produced by in vitro fertilization. At 6.5 h after insemination, the fertilized oocytes were cryopreserved by the simple vitrification method [32]. After warming, the oocytes can be readily used for microinjection or electroporation to edit the target gene using the TALEN or CRISPR-Cas9 system [32–36].

### Superovulation

Superovulation is a useful technique to obtain a large number of oocytes via the administration of hormones [37]. Ovulated oocytes are used for cryopreservation, in vitro fertilization, or mating to obtain fertilized oocytes in vivo. To induce superovulation, equine chorionic gonadotropin (eCG) and human chorionic gonadotropin (hCG) are administered routinely to female mice [38]. The average yield using the eCG and hCG method is 25 oocytes/female mouse [39]. In 2015, we refined the superovulation technique by the coadministration of inhibin antiserum (IAS) and eCG (IASe or ultrasuperovulation), which was able to produce more than 100 oocytes/female mouse [40]. IAS blocked the negative feedback of inhibin on the secretion of follicle-stimulating hormone (FSH), resulting in the production of excess levels of FSH and the promotion of follicular development [41, 42]. The coadministration of IAS and eCG was found to be effective in stimulating follicular development by endogenous and exogenous FSH. The ultrasuperovulation technique was helpful in reducing the number of oocyte donors and achieved a rapid and mass production of genetically engineered mice. With IASe treatment, 4-week-old C57BL/6 J female mice produced the largest number of oocytes at the age between 3 and 50 weeks [43]. The yield of ovulated oocytes using the IASe treatment was different between inbred mice (A/J: 24.9 oocytes/female; BALB/cByJ: 90.3 oocytes/female; C3HeJ: 52.0 oocytes/female; DBA/2 J: 68.8 oocytes/female; and FVB/NJ: 25.6 oocytes/female) and outbred mice (CD1: 33.7 oocytes/female) [44]. Among the highly immunosuppressed mouse (nonobese diabetic/Shi-scid IL2rγnull mouse), female mice aged 12 weeks produced the largest number of oocytes (70.0 oocytes/female) [45]. Therefore, the optimal age of female mice to induce ultrasuperovulation using IASe treatment also depends on the mouse strain.

### Cold storage of sperm

The cold storage of sperm is applicable to the shipment of genetically engineered mice as an alternative to the shipment of live animals [46]. The shipment of cold-stored sperm can be done easily using inexpensive shipment and avoids the risks of spreading infectious diseases and the escape or death of live animals during the shipment. Regarding the shipment of sperm, we collected the cauda epididymis in a preservation solution and shipped it in a cold-transport kit [46]. We observed that the fertilizing ability of cold-stored mouse sperm decreased in a time-dependent manner [47]. However, the preservation solution of Lifor perfusion medium and in vitro fertilization using MBCD and GSH were found to be effective in preventing the reduction of the fertilizing ability of cold-stored sperm [46, 48]. Furthermore, the addition of DMSO and quercetin to the preservation medium prolonged the storage period of cold-stored sperm for 10 days [49]. The cold-stored sperm could be cryopreserved and later used to recover animals by in vitro fertilization and by embryo transfer [50]. Today, we generally receive cold-stored sperm to produce embryos or live animals or to archive cryopreserved sperm in the CARD mouse bank. The new transport system using the cold-stored sperm facilitated the domestic and international transportation of mouse resources.

### Cold storage of two-cell embryos

The cold storage of two-cell embryos has also been found to be useful for the shipment of genetically engineered mice [51]. The transported two-cell embryos can be used to produce animals by embryo transfer at the receiving facility. An advantage of the cold-transport of embryos is that it is a simple procedure without the need for cryopreservation and avoids the potential risks involved in the shipment of live animals. The developmental ability of cold-stored embryos could decline in a time-dependent manner. The preservation M2 medium containing 1.5 mM NAC was found to prolong the storage period of cold-stored embryos for 4 days [51–53]. Cold-stored embryos can also be cryopreserved using the simple vitrification method [54]. The shipment of cold-stored embryos is practical for performing embryo transfer beyond the facility or when there is a lack of mice recipients on the date of embryo transfer.

### Mouse reproductive technology workshop

To share our knowledge and techniques of mouse reproductive technology with our community, we have been organizing the CARD Mouse Reproductive Technology Workshop in Japan and abroad since 2000 (Table 2). In this workshop, we provide lectures, demonstrate the latest techniques, and perform hands-on training on the preparation of glass pipettes, oocyte handling, sperm cryopreservation, cold storage of sperm, in vitro fertilization using fresh, frozen–thawed, and cold-stored sperm, oocyte washing and observation, cryopreservation of oocytes and in vitro fertilization using vitrified–warmed oocytes, two-cell embryo collection, embryo cryopreservation, cold storage of embryos, operation of vasectomized mice, surgery of embryo transfer, and nonsurgical transportation of embryos. More than 700 students have participated in our workshops. Owing to the prevailing coronavirus pandemic, we have postponed the hands-on training and plan to set up an online course to overcome the limitations of international travel. Moreover, we intend to update new techniques on our website regarding the online manual of mouse reproductive technology (http://card.medic.kumamoto-u.ac.jp/card/english/sigen/manual/onlinemanual.html).

**Table 2:** Venues of CARD Mouse Reproductive Technology Workshop

Venue	Region
CARD, Kumamoto University	Japan
Asahikawa Medical University	Japan
Central Institute for Experimental Animals (CIEA)	Japan
Shanghai Laboratory Animal Center (SLAC)	China
National Laboratory Animal Center (NLAC)	Taiwan
The National Institute for Food and Drug Control (NIFDC)	China
Biological Resource Centre at A*STAR	Singapore
National Centre for Biotechnology at Spanish National Research Council (CNB-CSIC)	Spain
Roswell Park Cancer Institute	USA
Korea Research Institute of Bioscience & BioTechnology (KRIBB)	Korea
Institute Pasteur	France
Texas A&M Institute of Genomic Medicine	USA
Jackson Laboratory	USA

## Conclusions

**Fig. 1:**
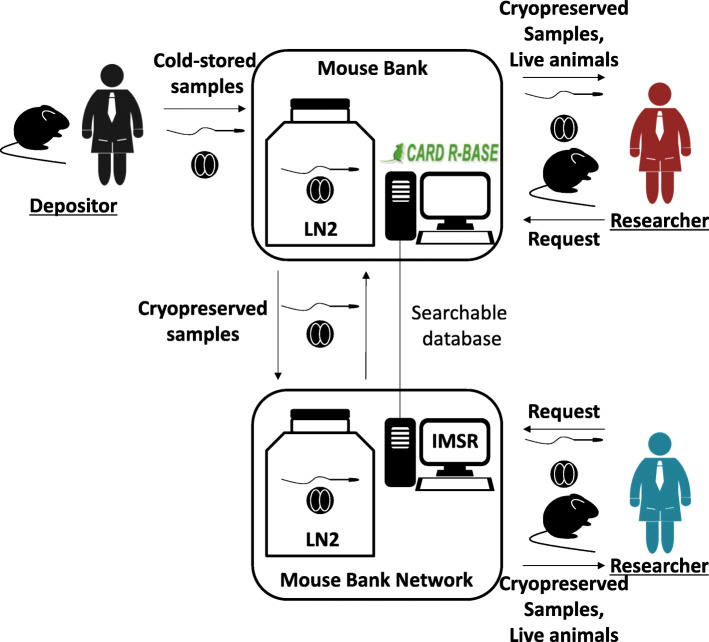
Application of advanced mouse reproductive technology in a mouse bank

The cryopreservation of mouse resources is an important strategy to accumulate valuable mouse characteristics useful for the scientific community. The optimal combination of reproductive technology will provide the best standards of cryopreserved mouse resources to researchers. We have provided a picture of the mouse bank system in Fig. 1. The advanced mouse bank system will provide a seamless archive and supply of mouse resources beyond facilities and countries. In addition, an international resource network will provide a robust research infrastructure to facilitate international collaborations. We have described the latest techniques of mouse reproductive technology in this review article. Details of the techniques can be mastered via the hands-on workshop or our online manuals. We hope that this review article would be helpful in improving the management and availability of mouse resources at your facility.

## Data Availability

Not applicable.

## Abbreviations

CARD: Center for Animal Resources and Development
IMSR: International Mouse Strain Resource
AMMRA: Asian Mouse Mutagenesis and Resource Association
MBCD: Methyl-β-cyclodextrin
GSH: Reduced glutathione
DMSO: Dimethyl sulfoxide
DAP213: 2 M DMSO, 1 M acetoamide, and 3 M propanediol
NAC: N-acetyl cysteine
TALEN: Transcription activator-like effector nuclease
CRISPR: Clustered regularly interspaced short palindromic repeats
Cas9: CRISPR-associated nuclease 9
eCG: Equine chorionic gonadotropin
hCG: Human chorionic gonadotropin
IAS: Inhibin antiserum
IASe: Inhibin antiserum and equine chorionic gonadotropin
FSH: Follicle-stimulating hormone
